# Chlorate of Potassa

**Published:** 1858-05

**Authors:** T. S. Weatherly

**Affiliations:** Montgomery, Alabama


ATLANTA

Medical and Surgical Journal.

VOL. III.] MAY, 1858. [No. IX.

### ORIGINAL COMMUNICATIONS.

### ARTICLE 1.

*Chlorate of Potassa.* By T. S. WEATHERLY, M. D., Montgomery, Alabama.

It seems that epidemics are the order of the day. We have epidemics in politics and religion, and our Profession is at times affected with an epidemic, called Cacœthes Scribendi.

Some few years since, this was manifested in the Southern Journals, by innumerable articles upon veratrum viride, dysentery and typhoid fever, and recently I see evidences of its again appearing in the form of chlorate of potassa. It is to this latter subject that I wish to call your attention. If I am not very much mistaken, I wrote the first article upon this subject, which you did me the honor to publish, about twelve months since. In that article it was recommended in Typhoid Fever, and other diseases of the mucous membranes.

I did not pretend to any originality, and so stated in that article. I commenced using it in Typhoid Fever about five years ago. I did so from my idea of its therapeutic action upon mucous membranes, and from the pathology of typhoid fever.

I was living at the time in a Typhoid Fever District, and was exceedingly anxious to find some remedy upon which I could depend. I was pleased with its effects, and have continued its use up to the present time. I think it very probable that other men may have used it in the same way, and for the same diseases, long before I did. But as I remarked above, I believe that my article was the first *publication* upon the subject on this side of the Atlantic, with probably the exception of Prof. Frost, of Charleston, and I think he did not use it in typhoid fever, though of that I am not certain.

It is a matter of very little importance, but you know the old adage—"give the devil his due."

